# Shape-tailoring and catalytic function of anisotropic gold nanostructures

**DOI:** 10.1186/1556-276X-6-547

**Published:** 2011-10-05

**Authors:** Thathan Premkumar, Kyungjae Lee, Kurt E Geckeler

**Affiliations:** 1 Department of Materials Science and Engineering, Gwangju Institute of Science and Technology (GIST), 1 Oryong-dong, Buk-gu, Gwangju 500-712, South Korea; 2 Department of Nanobio Materials and Electronics, World-Class University (WCU), Gwangju Institute of Science and Technology (GIST), 1 Oryong-dong, Buk-gu, Gwangju 500-712, South Korea

**Keywords:** gold, nanoparticles, detergent, catalytic activity, one-pot synthesis

## Abstract

We report a facile, one-pot, shape-selective synthesis of gold nanoparticles in high yield by the reaction of an aqueous potassium tetrachloroaurate(III) solution with a commercially available detergent. We prove that a commercial detergent can act as a reducing as well as stabilizing agent for the synthesis of differently shaped gold nanoparticles in an aqueous solution at an ambient condition. It is noteworthy that the gold nanoparticles with different shapes can be prepared by simply changing the reaction conditions. It is considered that a slow reduction of the gold ions along with shape-directed effects of the components of the detergent plays a vital function in the formation of the gold nanostructures. Further, the as-prepared gold nanoparticles showed the catalytic activity for the reduction reaction of 4-nitrophenol in the presence of sodium borohydride at room temperature.

## Background

Nanosized metal particles are of great interest and important for their applications in an ample diversity of areas such as catalysis, optical devices, nanotechnology, and biological sciences [1-4]. Among the nanoparticles of many metals, gold (Au) nanoparticles have gained much attention, because they have shown to be a technologically important material that can be potentially used in application such as catalysis [5,6], chemical sensors [7], in biological and medical areas [4,8], and for the miniaturization of electronic devices due to their unique optical and electrical properties [4,9-12]. Interestingly, these characteristic properties often depend critically not only on the particle size, but also on the particle shape [13-15]. Specially, the formation of aqueous dispersible nanoparticles has concerted many efforts in view of prospective biomedical applications and environmental effects connected with the use of organic solvents. Depending on the synthesis techniques and the kind of stabilizing and reducing agents, particles with various properties can be generated. While the size-controlled synthesis of gold nanoparticles (AuNPs) has been actively pursued, little has been done to shape manipulations of AuNPs in an aqueous medium, which could facilitate the various potential applications in the fields of physics, chemistry, biology, medicine, and materials science as well as their different interdisciplinary fields.

Though a number of preparative protocols have been attempted and introduced to control the shape of silver nanoparticles [16-20], analogous reports for AuNPs are comparatively few and are more recent. These include liquid crystal [21], templates [22], solution-based techniques [23,24], and others [25] guide to the fabrication of planar Au nanostructures with reasonable control over their optical properties. Further, it has been reported that AuNPs with controlled shapes were synthesized by introducing acetylacetone and several related ligands [26]. For example, hexadecylaniline was used for the synthesis of organically dispersible AuNPs, where the spontaneous reduction of aqueous HAuCl_4 _solution results in variable shapes of nanoparticles [27]. Recently, biosynthetic methods, an alternative to chemical synthetic procedures and physical processes, have been introduced to the formation of AuNPs by using plant extracts [28,29]. Also, an excellent shape-selective formation of single crystalline triangular AuNPs by using the extract of a lemongrass plant (*Cymbopogon flexuosus*) was reported [30,31]. In a break from tradition, which has hitherto relied on the use of either reducing agents such as NaBH_4_, N_2_H_4_, and/or external energies such as photochemical, microwave irradiation, and radiolysis in the synthesis of AuNPs in an aqueous solution, we have recently shown that neutral surfactants such as polysorbate 80 may be used to synthesize spherically shaped AuNPs of different sizes in an aqueous solution at different experimental conditions without utilizing any additional reducing agents and energies [32].

In this paper, we report the facile, one-pot, shape-selective synthesis of AuNPs in high yield by the reaction of an aqueous KAuCl_4 _solution with a commercially available detergent. We demonstrate that by altering the reaction conditions such as concentration of the reactants and temperatures, the percentage of Au nanostructures can be manipulated. It is considered that a slow reduction of the Au ions along with the shape-directed effects of the components of the detergent plays a vital function in the formation of the Au nanostructures. The approach introduced here does not need any harsh and toxic reducing agents and it requires no manipulative skills. Further, the as-prepared AuNPs showed the catalytic activity for the reduction reaction of 4-nitrophenol in the presence of sodium borohydride. An important aspect of nanotechnology concerns the development of experimental processes for the synthesis of nanoparticles of different chemical compositions, sizes, shapes, and controlled dispersity with facile approach and low cost. This method is facile and employs gentle reaction conditions in contrast to the conventional techniques using polymers or surfactants and harsh reductants.

## Results and discussion

**Figure 1:**
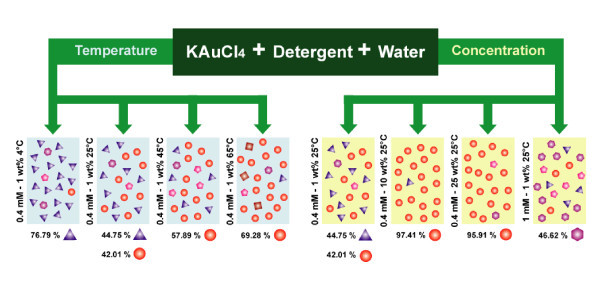
**Shape distribution of AuNPs**. Schematic illustration for the formation and shape distribution of AuNPs at ambient experimental conditions.

Gold nanoparticles were synthesized by mixing appropriate concentrations of solutions of AuCl_4_- and detergent at room temperature. The resulting solution was shaken for homogenization and kept at different temperatures (4°C, 25°C, 45°C, and 65°C) for the reaction to proceed. Different concentrations of reactants (metal ion and detergent) were also carried out, and the changes that occurred in the formation of nanoparticles were studied systematically. Final concentrations of the metal ion and detergent in each sample (expressed as the millimolar concentration of metal ion and weight percent concentration of detergent, respectively) as well as the experimental conditions and shape distributions of the AuNPs, are summarized in Figure 1.

**Figure 2:**
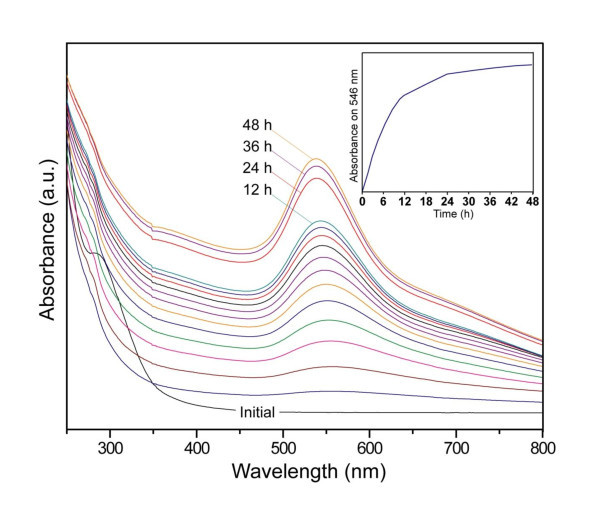
**UV-visible absorption spectra**. UV-visible absorption spectra during the reaction at room temperature of a sample prepared with 0.4 mM Au salt and 1 wt.% detergent. The inset shows the corresponding plasmon band intensity around 546 nm as a function of time.

Mixing an aqueous solution of 0.4 mM concentration of Au salt and detergent (1 wt.%) at room temperature led to the appearance of a pinkish color of the solution after about 5 h of reaction time, demonstrating the formation of AuNPs. The UV-visible (UV-vis) absorption spectrum recorded from this solution shows the characteristic surface plasmon resonance band of AuNPs [12] centered at 546 nm (Figure 2). The kinetics of formation of AuNPs was followed by UV-vis spectroscopy, and the spectra obtained are shown in Figure 2. It is observed that with the progress of the reaction, the absorbance intensity at 546 nm increases constantly with time, while the absorption peak of AuCl_4_- (initial) at about 290 nm disappeared suddenly after about 1 h of reaction time. Additionally, the surface plasmon absorption band undergoes a slight blue shift from 558 to 540 nm with the increase of time. This shift might be due to a decrease in the size of AuNPs formed. This band evolved with time, finally reaching a constant absorbance after 48 h and only slight increases in absorption occurred thereafter. Both the final absorbance value and the peak position remained constant even after storage for several weeks. To evidently display the reaction dynamics of the formation of AuNPs with time, the dependence of the absorption intensity of the AuNPs at around 546 nm with time is also shown (Figure 2, inset). As seen from this curve, the reaction was almost terminated after around 48 h.

**Figure 3:**
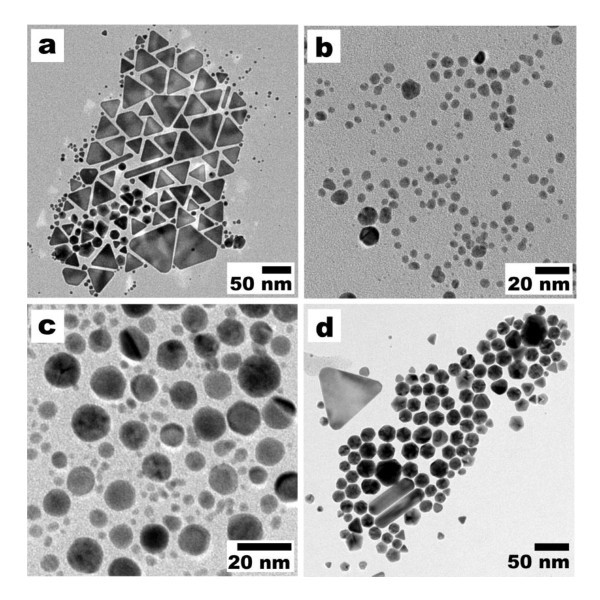
**TEM micrographs of AuNPs produced at different concentrations**. TEM micrographs of AuNPs obtained at room temperature with different concentrations of Au salt and detergent. (**a**) 0.4 mM, 1 wt.%, (**b**) 0.4 mM, 10 wt%, (**c**) 0.4 mM, 25 wt%, and (**d**) 1 mM, 1 wt%.

It was observed that the shapes of the nanoparticles were sensitive to the concentrations of the Au salt and detergent. By varying the concentrations of the reactants, we were able to produce nanocrystals of different shapes. In order to study the influence of concentration of the reactants with respect to the shape of the AuNPs, the reactions were performed at room temperature with 0.4 mM molar concentration of Au precursor; 1, 10, and 25 wt.% concentrations of detergent; and 1 mM and 1 wt.% concentrations of Au precursor and detergent, respectively (Table 1). Transmission electron microscopy (TEM) image (0.4 mM Au salt and 1 wt.% detergent, Figure 3a) showed that an almost equal percentage of particles had both triangular (44.75%) and spherical (42.01%) shapes. Worth to mention here is that an increase of the concentration of the detergent to 10 and 25 wt.%, while keeping the concentration of the Au precursor constant, led to a formation of Au nanospheres or spherical shape (Figure 3b,3c) AuNPs with >95% (see Table 1). In the recent reports, the metal plate-like structures have been obtained by using mild reaction conditions, which play a significant role in the nucleation and growth of anisotropic particles [23,31,33]. In the present case, the detergent acts in a dual role as reducing and protecting agent. Thus, it has to be considered that by raising the concentration of detergent, both the reduction rate and protecting action of the detergent solution increase. We anticipate that the high concentration (10 and 25 wt.%) of the detergent gave rise to an increase in the reduction rate that may influence the formation of spherically shaped AuNPs by increasing the rate of nucleation and growth. This may be the reason why the spherical AuNPs is favored while increasing the concentration of the detergent.

**Table 1:** Major shape distribution of AuNPs at different experimental conditions

Concentration of gold salt (mM)/detergent (wt.%)	Temperature (°C)	Shape distribution (%)			
		Triangular	Spherical	Pentagonal	Hexagonal
0.4/1	25	44.8	42.0	6.4	5.5
0.4/10	25	1.3	97.4	0.7	0.7
0.4/25	25	0.6	95.3	1.4	2.7
1/1	25	18.1	14.3	46.6	19.6
0.4/1	4	76.8	7.1	5.4	10.7
0.4/1	45	25.8	57.9	14.7	1.6
0.4/1	65	6.1	69.3	6.8	2.7

In one particular experiment, the Au precursor concentration was increased to 1 from 0.4 mM, which was used for the synthesis of triangle and spherical nanoparticles, and the final concentration between the Au precursor and detergent was maintained at 1 mM and 1 wt.%, respectively. Interestingly, the observation by TEM showed (Figure 3d) that approximately 47% of the particles had a projected hexagonal shape and a size range of 20 ± 5 nm. In addition to the hexagonal shapes, which formed the majority of the product, a small portion (approximately 18%) of pentagonal (sizes of 19 ± 5 nm), triangular, and spherical (each approximately 14%) particles were also commonly observed in the final products (Table 1).

**Figure 4:**
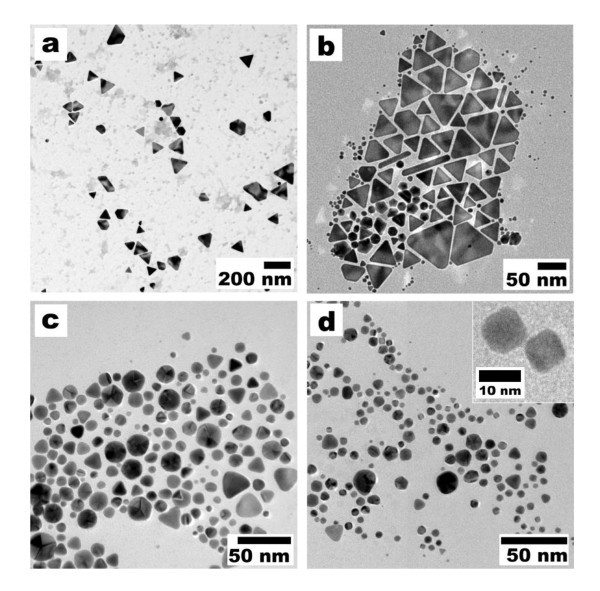
**TEM micrographs of AuNPs obtained at different temperatures**. TEM micrographs of a sample obtained with a concentration of 0.4 mM Au salt and 1 wt.% detergent at different temperatures: (**a**) 4°C, (**b**) 25°C, (**c**) 45°C, and (**d**) 65°C. The inset in (d) shows the representative image of Au nanocubes.

It is pertinent to mention that further shape control was noticed by simply varying the temperature of the reaction conditions, while keeping the concentration of the Au precursor and the detergent constant (0.4 mM and 1 wt.%). With the aim to analyze the effect of temperature on the shape of the AuNPs, four different temperatures (4°C, 25°C, 45°C, and 65°C) were selected, and the shape distribution of the AuNPs was confirmed from TEM measurements. Figure 4a,b,c,d shows the TEM micrographs of the reaction products corresponding to temperatures of 4°C, 25°C, 45°C, and 65°C, respectively. As can be perceived, the variation of temperature has a significant effect on the shape of the nanoparticles obtained.

**Figure 5:**
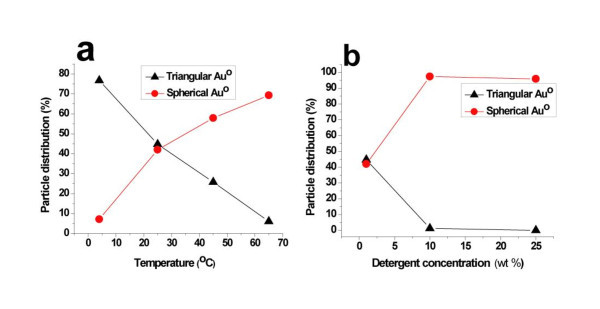
**Shape distribution of triangular and spherical AuNPs**. Graph showing the temperature (**a**) and detergent concentration (**b**) effects relative to the shape distribution of triangular and spherical AuNPs.

The TEM analysis clearly reveals the formation of triangular, spherical, and a smaller number of hexagonal and pentagonal nanoparticles. The careful analysis shows that at 4°C almost approximately 80% of the total nanoparticle population was owing to triangular or truncated triangular AuNPs (average size or edge length, approximately 80 nm). This is considerably higher than values reported previously [30]. As the temperature of the reaction condition was increased from 4°C to 25°C, the percentage distribution of the triangularly shaped particles decreased (44.75%) and the formation of spherically shaped (42%) nanoparticles increased (Figure 4b). The same tendency was observed with a further increase in temperature to 45°C (triangular shape, 26%; spherical shape, 58%) and 65°C (triangular shape, 6%; spherical shape, 70%). It is interesting to mention here that pentagonally (approximately 15%, Figure 4c) and cubicly shaped (approximately 15%, Figure 4d and inset) AuNPs were also observed at 45°C and 65°C, respectively, in addition to the aforesaid triangularly and spherically shaped nanoparticles. From the results observed, we conclude that the percentage formation of triangularly shaped AuNPs increased with decreasing the reaction temperature, while the percentage formation of spherically shaped particles increased with increasing the reaction temperature (Table 1), which can be explained by the higher reactivity of the detergent at a higher temperature. It has been recently reported [23,31] that slow reaction conditions play a vital role in the growth of metal plate-like structures or anisotropic particles of crystalline nature, which have been evidently observed in our studies. These results clearly show that by varying the reaction temperature and using the same concentration ratio of AuCl_4_- to detergent, the shape of AuNPs can be readily tuned. Interestingly, as the temperature increased, the reaction time for the formation of AuNPs decreased. The correlation between the reaction temperature or concentration and the particle shape distribution is shown in Figure 5. It markedly shows that the formations of triangularly shaped particles are inversely proportional to the temperature (Figure 5a) or concentration (Figure 5b), whereas the spherically shaped particles are directly proportional. The TEM images reveal that the as-prepared particles show a minimal polydispersity and are both well dispersed in the reaction medium and non-agglomerated.

**Figure 6:**
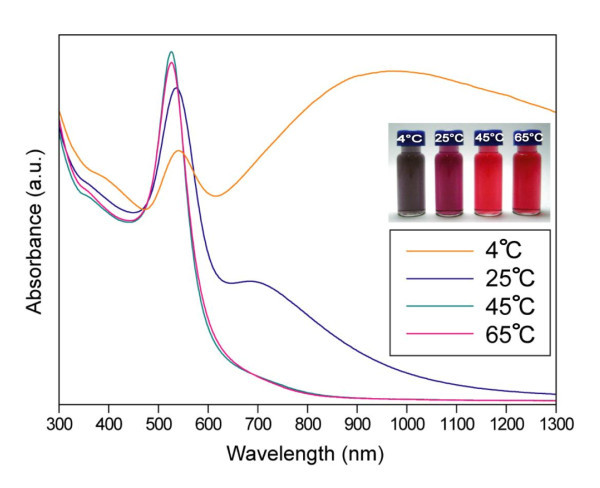
**UV-vis-NIR absorption spectra of AuNPs**. UV-vis-NIR absorption spectra of AuNPs observed during the reaction of Au salt (0.4 mM) with detergent (1 wt.%) at different temperature. The inset shows the photographs of the different nanoparticle solutions with color changes during the formation of AuNPs as a function of temperature.

It was found that AuNPs of different shapes clearly displayed different surface plasmon resonance [34]. It is well known that the triangular nanoparticles of Au exhibit two characteristic absorption bands referred to as the transverse (out-of-plane) absorbance, approximately concurring with the surface plasmon resonance of spherically shaped AuNPs, and the longitudinal (in-plane) surface plasmon resonance band, which is due to the strong edge length of the triangles [31]. The absorbance bands observed at 538 and 996 nm in the UV-vis-near-infrared (NIR) spectrum (Figure 6) are thus clearly due to the *out-of-plane *and *in-plane *surface plasmon resonance bands of the nanotriangles being formed at the reaction conditions at 4°C. Sharp peaks located at 526 nm were observed for the AuNPs synthesized at 45°C and 65°C, implying the formation of nanospherical shaped particles [32]. The UV-vis-NIR spectrum of the AuNPs obtained at 25°C resembles that of spherical nanoparticles (536 nm). The additional broader shoulder observed at 684 nm is most likely to arise from co-existing triangular AuNPs. The spectral features of the nanotriangles and spherical nanoparticles are fairly consistent with the TEM results as well as previous reports [30,31,35,36].

**Figure 7:**
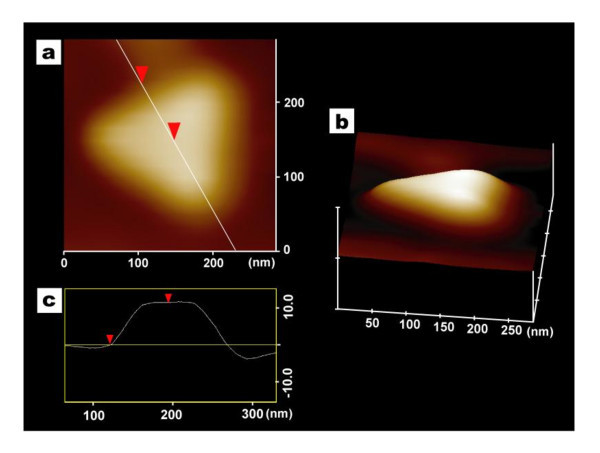
**AFM image of a single Au nanotriangle particle**. (a) Representative contact mode AFM image of a single Au nanotriangle particle obtained with the reaction of 0.4 mM Au salt and 1 wt.% detergent at 4°C temperature. (b) 3-D AFM micrograph of a single nanotriangle, and (c) topographic height analysis of Au nanotriangle.

Figure 7a shows an atomic force microscopy (AFM) image of individual or single Au nanotriangles synthesized by using the reaction of 0.4 mM Au precursor and 1 wt.% detergent at 4°C temperature. The topographic height analysis (Figure 7b) of a single Au nanotriangle and the surface profile plot (Figure 7c) show that the particle has a thickness of 11 nm and an edge length of approximately 140 nm.

Even though mechanisms for the formation of metal plate-like structures or anisotropic particles have been proposed according to the nature of the reaction conditions, it still remains a subject of controversy. However, it is generally accepted that the shape of an fcc nanocrystal is mostly decided by the ratio of the growth rate along the [100] versus the [111] direction [35]. It has been reported that the triangular and hexagonal nanoparticles bound by the stable [111] planes and the perfect cube bounded by less stable [100] planes [35]. Further, some authors proposed that the formation of the thin plate-like structures is due to the preferential adsorption of protecting agents such as some specific surfactants or polymers onto favored crystalline planes. It is obvious that the detergent, which contains complex functional compounds and (amine oxide, hydroxyl, and carboxylic) groups, plays the key role in the present case for the formation of differently shaped nanoparticles at ambient condition. We believe that the amine oxide and hydroxyl functional groups present in the detergent may facilitate the reduction process and the carboxylic groups may have an interaction with the surface of the AuNPs and in turn stabilize the AuNPs. The chances of influencing the shapes of AuNPs by other foreign materials are ruled out, since the detergent acts as both reducing and protecting agent and no additional agents or materials are added in the present system. Hence, we believe that the specific interaction between the detergent and the different surface planes of the AuNPs at ambient conditions could significantly increase the growth rate along the [100] direction and, in turn, reduce the growth rate along [111] plane, thus, favoring the formation of nanoparticles of triangular and hexagonal shape [33].

**Figure 8:**
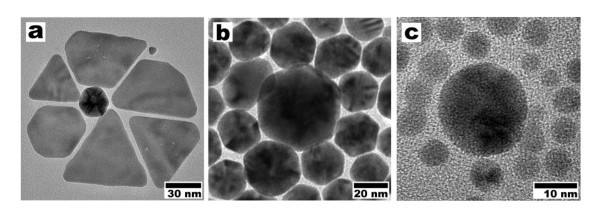
**Nanoflower and satellite-like structures of AuNPs**. (a) "Nanoflower" and (b, c) "satellite structures" of AuNPs.

Interestingly, occasionally we found in the TEM images that the nanoparticles of all the shapes self-assembled in such a way that they look like "nanoflowers" (Figure 8a) or "satellite-like structures" (Figure 8b,c). The TEM images of the system in the assembled state indeed show extended aggregates of two different-sized particles with a (big particle and small particle) periodicity. The nanoflower (Figure 8a) consists of single approximately 27-nm particle surrounded by six approximately 30- to 47-nm (of triangular or truncated triangular shape) particles, whereas the satellite structures (spherically and hexagonally shaped nanoparticles) comprise a big particle bounded by many small particles, as evidenced from the TEM images (Figure 8b,c). These self-assembled structures are significant, as these structures form the basis for a new type of building block that could be incorporated into multicomponent nanostructured materials [37,38].

**Figure 9:**
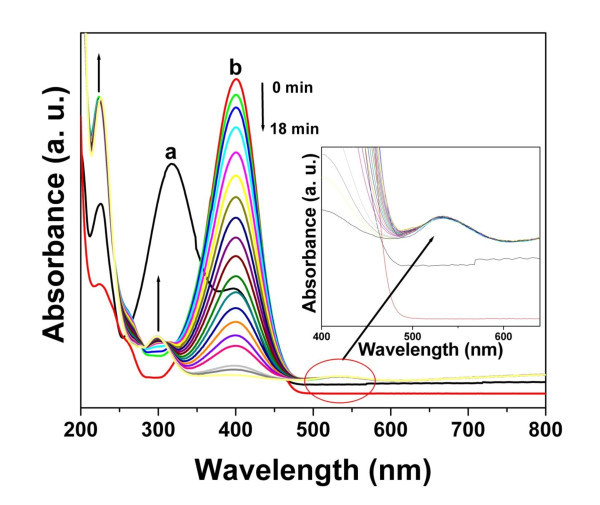
**UV-vis absorption spectra of the reduction of 4-nitrophenol in the presence of AuNPs**. UV-visible absorption spectra of (a) 4-nitrophenol and (b) successive UV-vis absorption spectra (1-min interval) of the reduction of 4-nitrophenol by NaBH_4 _in the presence of AuNPs prepared at 4°C. The inset shows the corresponding plasmon band intensity of AuNPs around 530 nm.

In order to investigate the catalytic activity of the as-prepared AuNPs at room temperature, we have carried out the reduction reaction of 4-nitrophenol by NaBH_4 _in the presence of as-prepared AuNPs. The change occurring in the solution was monitored visually as well as by UV-vis spectroscopy. It is evident that a typical absorption band of 4-nitrophenol (Figure 9a) undergoes a bathochromic shift from 316 to 400 nm, owing to the formation of the 4-nitrophenolate ion, after adding an aqueous solution of NaBH_4_. It is worth to mention here that the addition of AuNPs prepared at 4°C to the reaction medium showed a constant decrement of the 400-nm peak intensity (Figure 9b). Finally, it disappeared within 18 min with the concomitant appearance of new peaks at 298 and 231 nm (in addition to characteristic surface plasmon band of AuNPs in the 530-nm region; see Figure 9, inset), revealing the generation of 4-aminophenol. It was also observed visually that the light yellow color of the 4-nitrophenol turned to yellowish green rapidly after adding the aqueous solution of NaBH_4 _(formation of 4-nitrophenolate ion), which finally turned to almost colorless after 18 min upon addition of the as-prepared AuNPs, corroborating the formation of 4-aminophenol. It is pertinent to state that in the absence of the AuNPs, the peak due to the 4-nitrophenolate ion remained unaltered for a long time, showing the incapability of NaBH_4 _to reduce the 4-nitrophenolate ion to 4-aminophenol, even though it is known to be a strong reducing agent. This result clearly indicates that the as-prepared AuNPs catalyzed the reduction reaction, which is having a better catalytic activity than the reported citrate-reduced AuNPs (around 27 min) [39]. The catalytic activity of AuNPs is may be owing to proficient electron transfer from the BH_4_- ion to the nitro compound mediated by the nanoparticles.

**Figure 10:**
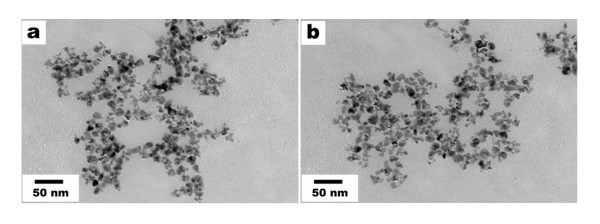
**TEM images of palladium nanoparticles**. TEM images of palladium nanoparticles obtained at (a) 45°C and (b) 65°C.

In addition, we investigated the efficiency of synthetic strategy to prepare other metal nanoparticles, and the groundwork outcomes observed for palladium nanoparticles are encouraging. To study the influence of the detergent for the production of the palladium nanoparticles, we carried out experiments at two different temperatures (45°C and 65°C). As a result, we found that it was possible to produce plate-like structures (Figure 10) by reacting the palladium precursor and the detergent in the aqueous medium. In both cases, plate-like structures were observed, and detailed studies on the influence of the different reaction parameters such as concentration as well as on the shape distribution are currently in progress.

## Conclusions

An easy and inexpensive, one-pot, shape-selective synthesis of AuNPs through the reaction of aqueous KAuCl_4 _with a commercially available detergent was developed. We proved that a commercial detergent can act as a reducing as well as the stabilizing agent for the synthesis of differently shaped AuNPs in an aqueous solution at an ambient condition. Hence, this route permits well-dispersed AuNPs to be obtained at room temperature, without employing reducing agents and external energy. Therefore, this approach is facile and inexpensive method for the shape-selective synthesis of AuNPs, even without any additional reagents and sophisticated equipment or facilities. The approach introduced here does not need any harsh and toxic reducing agents that might enhance the local concentration of any reagent in solution during addition. Thus, the synthetic process is thought viable to be readily integrated into a variety of systems, especially those that are relevant to biomedical applications, as it uses water as the solvent. It is noteworthy that the AuNPs with different shapes can be prepared by simply changing the reaction conditions. It is considered that a slow reduction of the Au ions along with the shape-directed effects of the components of the detergent plays a vital function in the formation of the Au nanostructures. Further, the as-prepared AuNPs showed catalytic activity for the reduction reaction of 4-nitrophenol in the presence of NaBH_4_, which has been established by UV-vis spectroscopy. This method is facile and employs gentle reaction conditions in contrast to the conventional techniques using polymers or surfactants and harsh reductants. The morphology and dimensions of the product were found to strongly depend on the reaction conditions such as concentration of the Au precursor, detergent, and temperature. This synthetic strategy has the potential to be a generalized process that can be extended readily to the synthesis of different kinds of metal plate-like structures. Studies in this direction are underway.

## Methods

### Chemicals

KAuCl_4 _as the precursor for the formation of AuNPs was obtained from Aldrich (St. Louis, MO, USA). Commercial detergent (Jayeonpong, brand name) from LG Household and Health Care, Seoul, South Korea and was used as both reducing and protecting agents. It consists of approximately 23% surfactants such as alcoholic (anionic), olefinic (anionic), and aminic (nonionic) and approximately 77% pine needles extracts, etc. 4-Nitrophenol (Junsei, Tokyo, Japan) and NaBH_4 _(Aldrich) were purchased and used without further purification.

### Characterization

The UV-visible absorption spectra were recorded on a Varian Cary 500 spectrophotometer (Varian, Inc., Palo Alto, CA, USA). The TEM was performed with a Philips T20ST instrument (Philips, Amsterdam, Netherlands). The TEM specimens were prepared by placing a few drops of sample solution on a copper mesh covered with a carbon film and allowing the solvent to evaporate at room temperature for overnight. The particle shape distributions were calculated by image analysis, always over more than 100 counts. The UV-vis-NIR spectroscopic measurements of the AuNPs prepared were analyzed on a JASCO model V-570 dualbeam spectrophotometer (JASCO, Easton, MD, USA). The AFM images of AuNPs were obtained in the contact mode on a Dimension™ 3100 Atomic Force Microscope (Digital Instruments, Veeco Metrology Group, Santa Barbara, CA, USA).

### Synthesis of gold nanoparticles

The AuNPs were prepared by the reduction of Au^3+ ^ions in an aqueous solution containing the detergent at room temperature. The procedure was quite easy and straightforward. In a typical experiment, 0.4 mM KAuCl_4 _was added to 10 mL of 1 wt.% aqueous solution of the detergent at room temperature, leading to the slow formation of AuNPs, as manifested by a pinkish coloration of the solution. No stirring was necessary after the solution was gently hand shaken (approximately 1 min) for homogenization. Therefore, the samples were left standing for the reaction to proceed at room temperature. The extent of the reaction depended on the concentration of detergent, while the time needed for its completion mainly depended on the concentration of the reactants and on the reaction temperature.

### Catalytic reduction of 4-nitrophenol

An aqueous solution of NaBH_4 _(1 mL, 15 mM) was mixed with 4-nitrophenol (1.7 mL, 0.2 mM) in a standard quartz cuvette. The light yellow color of the 4-nitrophenol was turned to yellowish green due to the formation of 4-nitrophenolate ion. An aliquot of AuNPs prepared at 4°C (0.3 mL) was added to the resulting solution, and the time-dependent absorbance spectra were recorded with a time interval of 1 min in the scanning range of 200 to 800 nm at room temperature.

## Abbreviations

Au: gold; AuNPs: gold nanoparticles; TEM: transmission electron microscopy; AFM: atomic force microscopy.

## Competing interests

The authors declare that they have no competing interests.

## Authors' contributions

TP conceived the study, conducted the experiments, performed characterization, analyzed the data, interpreted the results, and wrote the manuscript. KL helped in the technical support for experiments and characterization. KEG designed the experiments, supervised, and corrected the manuscript. All the authors read, discussed the results, and approved the final manuscript.
